# The Factors and Pathways Regulating the Activation of Mammalian Primordial Follicles *in vivo*

**DOI:** 10.3389/fcell.2020.575706

**Published:** 2020-09-30

**Authors:** Yao Chen, Weina Yang, Xu Shi, Chenlu Zhang, Ge Song, Donghui Huang

**Affiliations:** ^1^Institute of Reproduction Health Research (Institute of Family Planning Research), Tongji Medical College, Huazhong University of Science and Technology, Wuhan, China; ^2^NHC Key Laboratory of Male Reproduction and Genetics, Family Planning Research Institute of Guangdong Province, Guangzhou, China

**Keywords:** primordial follicle, activation, signaling pathways, factors, mechanism

## Abstract

Mammalian ovaries consist of follicles as basic functional units. Each follicle comprised an innermost oocyte and several surrounding flattened granulosa cells. Unlike males, according to the initial size of the primordial follicle pool and the rate of its activation and depletion, a female’s reproductive life has been determined early in life. Primordial follicles, once activated, will get into an irreversible process of development. Most follicles undergo atretic degeneration, and only a few of them could mature and ovulate. Although there are a lot of researches contributing to exploring the activation of primordial follicles, little is known about its underlying mechanisms. Thus, in this review, we collected the latest papers and summarized the signaling pathways as well as some factors involved in the activation of primordial follicles, hoping to lead to a more profound understanding of the cellular and molecular mechanisms of primordial follicle activation.

## Introduction

It is universally accepted that the total population of primordial follicles within the postnatal mammalian ovary will not increase and be used up gradually (McGee and Hsueh, 2000; Maidarti et al., 2020). Primordial follicles are the reproductive units of the mammalian ovary that are composed of diplotene oocytes surrounded by a layer of flattened granulosa cells (Adhikari and Liu, 2009; Hsueh et al., 2015). They remain in a dormant phase until being recruited into the growing pool *via* a process known as primordial follicular activation (Lee and Chang, 2019; Wright et al., 2020). Physiologically, only a limited number of primordial follicles are activated to enter the growing follicle pool each wave (Hsueh et al., 2015; Zhang and Liu, 2015). The pool of primordial follicles drops sharply with the increase of a woman’s age, especially after 35 years (Hansen et al., 2008; Lew, 2019). When about 1,000 primordial follicles remain, this can lead to fertility cessation and the onset of menopause (Hansen et al., 2008; Ford et al., 2020). Therefore, the number of primordial follicles in the ovary determines the female reproductive ability (Oktem and Urman, 2010; Findlay et al., 2015).

Primordial follicle activation is characterized by the robust growth of oocyte and morphological changes of primordial follicle granulosa cells (pfGCs), i.e., the flattened pfGCs differentiate into cuboidal granulosa cells and then begin to proliferate (Liu et al., 2006; Hsueh et al., 2015). However, activation is irreversible. If the activated follicles are not selected for completing the process (culminating in ovulation), those follicles will undergo atresia at some stage of their maturation (John et al., 2008), which is a blow for infertile women with a diminishing ovarian reserve. But a global activation of all primordial follicles inevitably causes early exhaustion of the follicle pool and premature ovarian failure (POF) (Kawamura et al., 2016; Kallen et al., 2018). Given that dormant primordial follicles are the most abundant fertility reserve in female mammals and could be found in women with various ovarian diseases, using them for clinical treatment of female infertility is an excellent idea (Song et al., 2015; Zhang and Liu, 2015; Park et al., 2020). A new infertility treatment, called *in vitro* activation (IVA), was developed, which enables POF patients to conceive using their own eggs by the activation of residual dormant follicles (Kawamura et al., 2013, 2016). The birth of a healthy baby with the use of phosphatase and tensin homolog deleted on chromosome 10 (PTEN) inhibitors is a compelling evidence (Kawamura et al., 2013).

However, research on the activation of human primordial follicles is hindered due to limited access to tissue samples. Therefore, studies on the activation of primordial follicles have been largely performed in mice to assess its factors and pathways. Here, we will introduce the intracellular signaling mechanisms important for primordial follicle activation from the dormant state in mammals.

## Signal Pathways of Primordial Follicle Activation in Oocytes

### PI3K–Akt–FOXO3 Signaling Pathway in Oocytes

#### PI3K Signaling

Phosphoinositide 3-kinase (PI3K) signaling, a classic signaling pathway, is fundamental for regulating cell proliferation, survival, migration, as well as metabolism and consists of various signaling molecules including kinases, phosphatases, and transcription factors that establish cascades of intracellular signaling (Blume-Jensen and Hunter, 2001; Cantley, 2002; Zheng et al., 2012). In fact, an earlier study has reported that transient treatment of PI3K stimulators has been found to activate primordial follicles in neonatal mouse ovaries and in human ovarian cortical tissues (Li et al., 2010; Sun et al., 2015; Maidarti et al., 2020). In another experiment using PI3K stimulators as infertility treatment, one healthy baby was delivered out of 13 attempts (Kawamura et al., 2013). These results suggest that PI3K is a significant factor in the activation of primordial follicles.

In cells, the main function of PI3K is to phosphorylate phosphatidylinositol-4,5-bisphosphate (PIP2) to produce phosphatidylinositol-3,4,5-triphosphate (PIP3) at the intracellular membrane, whereas PTEN prevents the conversion of PIP2 to PIP3 (Cantley, 2002; Zhang et al., 2019). PIP3, if accumulated in the oocyte, could stimulate the phosphorylation of serine/threonine protein kinase (Akt) (Kim and Kurita, 2018) and increase the nuclear exclusion of forkhead box O3 (FOXO3), thereby triggering primordial follicle activation in humans and mice (John et al., 2008; Kawamura et al., 2013; Takeuchi et al., 2019). When unphosphorylated, FOXO3 will relocate to the nucleus and function as a transcription factor, leading to apoptosis and cell cycle arrest (Liu et al., 2006).

FOXO3, a downstream effector of PI3K signaling, will shuttle from the nucleus to the cytoplasm of mouse oocytes when PI3K signaling is activated in the oocytes (Ezzati et al., 2015; Lee and Chang, 2019). It is highly expressed in mouse primordial follicles, and its expression level would be declined in primary and later-growing follicles, indicating that FOXO3 relates to the activation of primordial follicles (Liu et al., 2007). To prove this, *FOXO3* was deleted from the oocytes of mouse primordial follicles, causing the global activation of dormant primordial follicles and the mutant female mice becoming infertile in young adulthood because of follicle depletion (Castrillon et al., 2003; Hosaka et al., 2004; Gallardo et al., 2008). In contrast, when the oocyte-specific *FOXO3* was overexpressed in mice, the mice showed infertility caused by the retardation of oocyte growth and follicular development (Liu et al., 2007), indicating that FOXO3 suppressed the activation of ovarian follicles (Cui et al., 2019). Furthermore, constitutively active *FOXO3* in oocytes could preserve the ovarian reserve in mice (Pelosi et al., 2013).

#### Molecules Regulating the PI3K Signaling Pathway

##### PTEN

PTEN, a lipid phosphatase, converts PIP3 back to PIP2 and thus acts as a negative regulator of PI3K signaling (Cantley, 2002). To explore the relationship between PTEN and follicular activation, *PTEN* was deleted from the oocytes of primordial follicles in mice, causing the entire primordial follicle pool to be activated prematurely and causing follicular depletion in young adulthood (Reddy et al., 2008). Meanwhile, the level of phospho-Akt was elevated in mutant mice (Reddy et al., 2008). Other studies using a PTEN inhibitor have also shown the activation of dormant primordial follicles due to enhancing PI3K signaling in mammals (Adhikari et al., 2012; Maidarti et al., 2019); thus, a healthy baby has been born from a primordial follicle of a woman with POF (Kawamura et al., 2013). These results suggest that PTEN is essential for maintaining the dormancy of the primordial follicle pool and most likely works through influencing PI3K signaling in mammals (Hu et al., 2018; Takeuchi et al., 2019).

##### Cell division cycle 42

Cell division cycle 42 (CDC42) is a member of the Rho GTPase family and is crucial in controlling multicellular functions including actin cytoskeletal dynamics, membrane trafficking, transcription, and cell cycle control (Duquette and Lamarche-Vane, 2014; Pérez et al., 2020). Previous studies have revealed that CDC42 is essential for meiotic maturation and might activate the PI3K pathway in mouse oocytes by regulating PTEN and PIP3 (Li et al., 2003; Na and Zernicka-Goetz, 2006). Recently, Yan et al. (2018) suggested that oocyte CDC42 controls follicle activation by activating PI3K signaling directly and downregulating PTEN expression in the oocytes of mouse dormant primordial follicles. They reported that CDC42 expression increases in oocytes during the activation of primordial follicles and that silencing of *CDC42* expression significantly suppressed primordial follicle activation. Most importantly, it revealed that PI3K signaling is activated through the binding of the CDC42 active form to the phosphatidylinositol-4,5-bisphosphate 3-kinase catalytic subunit beta, also called p110β, which is in accordance with previous idea (Fritsch et al., 2013).

**FIGURE 1:**
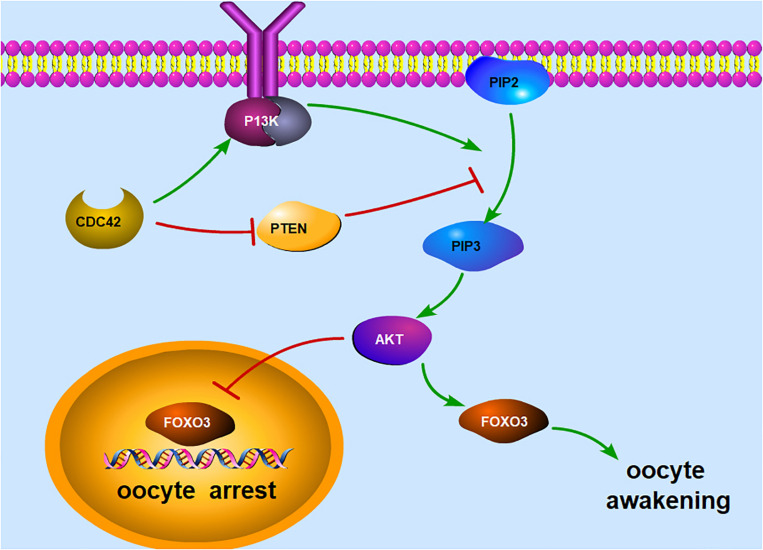
Illustration of the PI3K signaling pathway in an oocyte. The main function of PI3K is to phosphorylate PIP2 to produce PIP3 at the intracellular membrane, whereas PTEN prevents the conversion of PIP2 to PIP3. PIP3, if accumulated in the oocyte, could stimulate the phosphorylation of Akt and increase the nuclear exclusion of FOXO3, thereby triggering oocyte awakening. When unphosphorylated, FOXO3 will relocate to the nucleus and function as a transcription factor, leading to oocyte arrest. In addition, CDC42 can activate the PI3K signaling pathway by activating PI3K directly and downregulating PTEN expression. *PI3K*, phosphoinositide 3-kinase; *PIP2*, phosphorylate phosphatidylinositol-4,5-bisphosphate; *PIP3*, phosphatidylinositol-3,4,5-triphosphate; *PTEN*, phosphatase and tensin homolog deleted on chromosome 10; *Akt*, serine/threonine protein kinase; *FOXO3*, forkhead box O3; *CDC42*, cell division cycle 42.

In a word, these results suggest that the PI3K–Akt–FOXO3 signaling pathway plays an important role in maintaining the quiescence of primordial follicles in mammals (Figure 1). Loss of function of any of the inhibitory molecules for follicular activation, including PTEN and FOXO3, leads to a premature and irreversible activation of the primordial follicle pool (Adhikari and Liu, 2009).

### mTORC1–S6K1–rpS6 Signaling in Oocytes

#### mTORC1–S6K1–rpS6

The mammalian target of rapamycin (mTOR) is a highly conserved serine/threonine kinase that regulates cell growth, metabolism, survival, and migration, which modulate processes such as protein synthesis, ribosome biogenesis, and autophagy (Laplante and Sabatini, 2012; Yurube et al., 2020). PI3K/Akt not only localizes FOXO3 in the cytoplasm but also could activate mTOR signal. The PI3K/Akt/mTOR pathway is an intracellular signaling pathway important in regulating the cell cycle (Tewari et al., 2019). mTOR exists in two forms: mTORC1, which is sensitive to rapamycin, and mTORC2, which is not. The mTOR1 inhibitor rapamycin could suppress primordial follicle development and maintain follicle pool size in mice (Yorino and Kawamura, 2020). mTORC1 in oocytes plays an indispensable part in the activation of primordial follicles in the mouse ovary (Tong et al., 2013).

It was also found that the driving force behind mTORC1 signaling is the activated S6 kinase beta-1 (S6K1)–ribosomal protein S6 (rpS6) signaling that promotes protein translation and ribosome biogenesis in mouse oocytes (Reddy et al., 2010; Lee et al., 2012). S6K1 is the main substrate of mTORC1 and is phosphorylated by mTORC1 (Ahmed et al., 2019). S6K1 can also be activated by phosphoinositide-dependent kinase-1 (PDK1) (Gao et al., 2019), which may explain why the inhibition of mTORC1 signaling in mouse oocytes by deleting the regulatory-associated protein of mTOR (RPTOR) has no impact on follicular development and female fertility (Gorre et al., 2014). After being phosphorylated, S6K1 activates rpS6, which is essential for protein translation. Whether it is an incompetence of rpS6 in oocytes by deleting *PDK1* or deleting the *rpS6* gene, this would result in an accelerated loss of primordial follicles due to atresia, indicating that the mTORC1–S6K1–rpS6 cascade is indispensable for maintaining the survival of mouse primordial follicles (Reddy et al., 2009; Adhikari et al., 2010).

#### Molecules Regulating the mTOR Signaling Pathway

In cells, the activity of mTORC1 is negatively regulated by a heterodimeric complex consisting of tuberous sclerosis complex 1 (TSC1) and tuberous sclerosis complex 2 (TSC2) (Hansmann et al., 2020). The TSC1–TSC2 complex inhibits mTORC1 through a GTPase-activating protein domain located in TSC2, and TSC2 must be stabilized by TSC1 to protect it from ubiquitination and degradation (Chong-Kopera et al., 2006). In mutant mice lacking the *TSC1* in oocytes, the entire pool of primordial follicles is activated prematurely, and the disruption of *TSC2* in oocytes has shown an identical outcome (Adhikari et al., 2009, 2010). Furthermore, liver kinase B1 (LKB1), a multifunctional serine/threonine kinase secreted from mouse oocytes, can inhibit mTORC1 by phosphorylating TSC2 (Jiang et al., 2016).

### p27^Kip1^–CDK Signaling in Oocytes

p27^kip1^ is a member of the Cip/Kip family of cyclin-dependent kinase (CDK) inhibitors. It is a general CDK inhibitor whose specific late G1 destruction allows the progression of the cell across the G1/S boundary (Gonçalves, 2018). Rajareddy et al. (2007) showed that p27^kip1^ was expressed in the nuclei of mouse dormant oocytes and that primordial follicles in *p27*^kip1^-deficient mice were overactivated, resulting in POF. Hirashima et al. (2011) demonstrated that p27^kip1^ negatively regulated mouse primordial oocyte growth and that the knockdown of *p27*^kip1^ led primordial oocytes to enter the growth phase *in vitro*. These results indicated that p27^kip1^ plays a key role in preventing primordial follicle activation prematurely (Adhikari et al., 2009). But it is unknown for the regulation of p27^kip1^ –CDK signaling in oocytes. Jang et al. (2017) identified three putative FOXO3 binding elements in the mouse *p27*^kip1^ gene and found that FOXO3 could bind strongly to the putative binding site P2. Furthermore, cisplatin suppressed the binding affinity of FOXO3 for the putative elements in the *p27*^kip1^ promoter, and co-treatment with melatonin and ghrelin rescued FOXO3 binding to the P2 site of the *p27*^kip1^ promoter (Jang et al., 2017). Therefore, the p27^kip1^–CDK signaling in oocytes may be regulated by the PI3K–Akt–FOXO3 signaling pathway.

## Signaling Pathway of Primordial Follicle Activation in Granulosa Cells

### Activation of Primordial Follicles Through pfGCs

The growth of oocyte follows the proliferation and differentiation of pfGCs, which implies that pfGCs might be predominant in regulating the activation of primordial follicles (Zhang and Liu, 2015). However, a different view holds that oocytes govern follicular activation (Eppig et al., 2002; Adhikari and Liu, 2009; Yan et al., 2019). It is still a mystery whether pfGCs develop first to activate oocytes or not. But, until recently, modern research using genetically modified mouse models discovered a relatively complete signal transduction pathway in pfGCs to drive the development of oocytes (Zhang et al., 2014).

#### mTORC1 Signaling in pfGCs

Just as mTORC1 does in oocytes, mTORC1 in pfGCs also plays a key part in the activation of primordial follicles. RPTOR is the key component of the mTORC1 pathway in mouse pfGCs (Zhang et al., 2014). Zhang et al. (2014) discovered that the deletion of RPTOR in pfGCs caused the flattened pfGCs fail to differentiate into cuboidal granulosa cells, and compared to the control group, most of the oocytes in the mutant ovaries remained in a quiescent state in primordial follicles and eventually die out in adulthood. This result suggests that the deletion of RPTOR in pfGCs could suppress follicular activation and prevent the awakening of dormant oocytes (Zhang et al., 2014). In mouse ovaries with deletion of *TSC1*, all flattened pfGCs had differentiated into cuboidal granulosa cells and caused the premature awakening of all dormant oocytes and the activation of all primordial follicles (Adhikari et al., 2010). The same phenomenon also occurred in the ovaries that use the mTORC1 inhibitor rapamycin (Zhang et al., 2014). These results showed that elevated mTORC1 signaling in pfGCs could activate primordial follicles and that pfGCs are not only supporting the survival of dormant oocytes but are also determining their fates (Zhang et al., 2014).

#### KITL–KIT Signaling Is the Bridge Between pfGCs and the Dormant Oocytes

The KIT ligand, KITL, also called stem cell factor (SCF), is a receptor protein tyrosine kinase (Martinez-Anton et al., 2019). KITL plays important roles in primordial follicle activation, oocyte growth and survival, and granulosa cell proliferation in mammals (Tan et al., 2017; Adib et al., 2019; Islam et al., 2020). In mammalian follicles, KITL is produced by pfGCs (Vanderhyden, 2002; Jin et al., 2005), whereas KIT is expressed on the surface of oocytes (Driancourt et al., 2000). A previous study has demonstrated that the communication between mouse oocytes and the surrounding granulosa cells mainly depends on KIT and KITL (Driancourt et al., 2000). Zhang et al. (2014) found that active mTORC1 signaling enhances the expression of KITL in mouse pfGCs and that KITL–KIT signaling serves as the bridge between the pfGCs and the dormant oocytes for activating primordial follicles. Besides, PI3K signaling is one of the important downstream pathways activated by KITL–KIT in mice (Driancourt et al., 2000). KITL secreted from pfGCs binds to the KIT receptor on the oocyte surface, resulting in the phosphorylation of KIT Y719 and the subsequent activation of mouse oocyte PI3K signaling (Zhang et al., 2014). However, the mTORC1–KITL/KIT cascade is just a tip of the iceberg in the whole complex signaling pathways that associate pfGCs with the oocyte. More research is needed to figure out whether there are other factors involved between mTORC1 signaling and KITL/KIT.

### Activation of Primordial Follicles Through the HIPPO Signaling Pathway

#### Hippo Signaling

The Hippo signaling pathway is conserved in metazoan animals and is essential for organ size control (Halder and Johnson, 2011; Kwon et al., 2013; Gao et al., 2020). Hippo signaling consists of several negative growth regulators acting in a serine/threonine kinase cascade that eventually phosphorylates and inactivates key Hippo signaling effectors, Yes-associated protein (YAP)/transcriptional coactivator with PDZ binding motif (TAZ). When Hippo signaling is disrupted, decreases in the phosphorylated YAP (pYAP) increase the nuclear YAP levels, leading to the increased expression of downstream immediate early gene (CCN) growth factors and baculoviral inhibitors of apoptosis repeat containing (BIRC) apoptosis inhibitors (Kawashima and Kawamura, 2018). These CCN proteins, in turn, stimulate cell growth, survival, and proliferation (Kwon et al., 2013).

At present, there are a lot of ovarian diseases causing infertility, such as primary ovarian insufficiency (POI) and polycystic ovarian syndrome (PCOS). In the clinical setting, therapies for those patients who want to be pregnant include ovarian wedge resection (Andrews, 1952) and “drilling” by diathermy or laser due to their function of promoting follicle growth (Farquhar et al., 2012). Furthermore, extensive primordial follicle activation was observed after transplantation in marmoset, bovine, and human tissues (Gavish et al., 2018). Recently, a research found that the underlying mechanisms of these disruptive procedures, which have confused scientists for decades, were linked to the increased actin polymerization and, thus, the disruption of ovarian Hippo signaling (Kawamura et al., 2013). Fragmentation of the mammalian ovarian cortex into small cubes changed the cytoskeletal actin dynamics and induced the disruption of the Hippo signaling pathway, leading to the production of CCN growth factors and anti-apoptotic BIRC (Kawashima and Kawamura, 2018).

#### Activation of Primordial Follicles Through the HIPPO Signal

The expression of the core components of the Hippo pathway changes during mouse follicular development, especially before and after primordial follicle activation *in vitro*, which may be related to the significant decrease of the ratio of pYAP1/YAP1 (Sun et al., 2015). Kawamura et al. (2016) fragmented ovaries from juvenile (day 10) mice and grafted under kidney capsules for 5 days. The results showed that the percentages of the late secondary and antral/preovulatory follicles also increased, accompanied by decreases in primordial follicles. These results are in accordance with clinical therapeutic outcomes wherein human ovarian fragmentation could induce follicle growth, including the activation of primordial follicles (Kawamura et al., 2013). They discovered transient increases in the ratios of F-actin to G-actin after ovarian fragmentation and decreases in the pYAP levels as well as in the pYAP-to-total YAP ratios, suggesting Hippo signaling disruption (Kawamura et al., 2013). Masciangelo et al. (2020) confirmed that the Hippo pathway was involved in human follicle activation and suggested a link between the PI3K and Hippo pathways, with both contributing to the phenomenon of follicle burnout. Recently, Kawamura et al. (2020) used the drug-free *in vitro* activation approach to treat patients with poor ovarian response and retrieved more oocytes. Although it has been known that Hippo signaling exerts some effect on the initiation of primordial follicles, further research is needed to elucidate its specific mechanism.

## Factors Influencing the Activation of Primordial Follicles

There are multiple factors involved in the control of primordial follicle activation, and their functions are varied. Some factors promote primordial follicle activation while others inhibit it. There is a balance between the activation and maintenance of the primordial follicle pool. In short, these factors together maintain a normal and continuous follicular development process.

### Spermatogenesis- and Oogenesis-Specific Basic Helix-Loop-Helix-Containing Protein 1

Spermatogenesis- and oogenesis-specific basic helix-loop-helix-containing protein 1 (Sohlh1) is a germ cell-specific gene that encodes the basic helix-loop-helix transcriptional regulator that is essential in oogenesis and spermatogenesis (Suzuki et al., 2013). *Sohlh1* is specifically expressed in germ cell cysts and oocytes of primordial follicles in female mice, and *Sohlh1*-null mice are sterile due to a defect of follicle development during the primordial-to-primary follicle transition (Pangas et al., 2006). In *Sohlh1*^–/–^ mice, only primordial follicles and empty follicles can be observed in ovaries from 0.5 to 23.5 days post-parturition, and primordial follicle activation was not observed at 7.5 days post-parturition (Liu et al., 2019). The possible mechanism is that the absence of Sohlh1 may affect the activation of primordial follicles *via* the downregulation of KIT and the inhibition of the KIT/PI3K/Akt pathway (Liu et al., 2019). Compared with its expression in mice, Sohlh1 exhibits a much more extensive expression pattern in human tissues and is mainly located in the oocytes of primordial and primary follicles, granular cells, and theca cells of secondary follicles (Zhang et al., 2015).

### Newborn Ovary Homeobox Gene

Newborn ovary homeobox gene (NOBOX) is an oocyte-specific homeobox gene that plays a key role in controlling the process of primordial follicle activation (Bayne et al., 2015). *NOBOX* is expressed in primordial and growing oocytes in the ovaries of mice (Suzumori et al., 2002) and humans (Huntriss et al., 2006). The lack of *NOBOX* could accelerate postnatal oocyte loss and abolish the transition from primordial to growing follicles in mice (Rajkovic et al., 2004; Belli et al., 2013). The expression of *FOXO3* mRNA is significantly downregulated in the *NOBOX*-null mouse ovary (Choi et al., 2007), which indicates that NOBOX could regulate the activation of primordial follicles through the modulation of FOXO3. Furthermore, several *NOBOX* mutations have been reported to be associated with POI (Ferrari et al., 2016; Li et al., 2017).

### LIM Homeobox 8

LIM homeobox 8 (Lhx8) is a member of the LIM homeobox transcription factor family and is highly conserved (Zhou et al., 2015). Choi et al. (2008) showed that Lhx8 is expressed preferentially in the oocytes and germ cells within the mouse ovary and that *Lhx8*^–/–^ ovaries fail to maintain the primordial follicles, and the transition from primordial to growing follicles does not occur. Ren et al. (2015) found that the conditional deficiency of *Lhx8* in the oocytes of mouse primordial follicles leads to a massive primordial oocyte activation and partly functions by interacting with the PI3K signaling pathway indirectly. These results indicated that Lhx8 is essential for the maintenance of primordial follicles.

### Forkhead Box L2

Forkhead box L2 (FOXL2) is a member of the forkhead box superfamily of transcriptional factors that possess a DNA binding domain (Jin et al., 2020). Zheng et al. (2014) showed that FOXL2 is expressed in the pfGCs in mouse primordial follicles and in the granulosa cells in growing follicles. In *FOXL2*-null mice, ovarian granulosa cells do not complete the squamous-to-cuboidal transition, leading to the absence of secondary follicles and to oocyte atresia (Schmidt et al., 2004). Furthermore, nearly all primordial follicles have already initiated folliculogenesis 2 weeks after birth (Schmidt et al., 2004). In humans, *FOXL2* transcripts were less abundant in the granulosa cells from primary follicles compared to the granulosa cells from primordial follicles, providing preliminary evidence that *FOXL2* downregulation between these stages may coincide with a role in primordial follicle activation (Ernst et al., 2018). FOXL2 is highly expressed in the ovaries and the eyelids, and autosomal-dominant germline mutations in *FOXL2* cause blepharophimosis, ptosis, and epicanthus inversus syndrome (BPES) manifested by an eyelid malformation and POI (Crisponi et al., 2001). As can be seen above, FOXL2 is a key factor in regulating the activation of primordial follicles through initiating pfGCs.

### Bone Morphogenetic Proteins

Bone morphogenetic proteins (BMPs) constitute the largest subdivision of the transforming growth factor-β (TGF-β) family of ligands (Lowery and Rosen, 2018). There is accumulating evidence indicating that BMP4 and BMP7 are essential for the primordial-to-primary follicle transition (Lee et al., 2001; da Cunha et al., 2017). BMP4 and BMP7 are expressed in ovarian stromal cells and/or theca cells and have recently been implicated as positive regulators of the primordial-to-primary follicle transition (Knight and Glister, 2006).

BMP4-treated rat ovaries had a higher number of developing primary follicles and fewer arrested primordial follicles than the untreated controls (Nilsson and Skinner, 2003). BMP4 also promotes an increase in the diameters of the follicles and oocytes of primary and secondary follicles in an *in vitro* bovine ovary culture (da Cunha et al., 2017). Furthermore, the numbers of primary follicles were lower and those of primordial follicles were higher in anti-BMP-4-treated mouse ovaries compared to control ovaries (Tanwar et al., 2008). Gremlin1 and Gremlin2, as inhibitors of BMP4, have been demonstrated to reverse the effect of BMP4 to stimulate rat primordial follicle transition (Nilsson et al., 2014). When *Gremlin1* was knocked out, the mice died within 48 h after birth, and researchers discovered reduced oocyte numbers and a delayed primordial follicle development (Myers et al., 2011). These results indicated that BMP4 promotes primordial follicle development. However, it is interesting to note that, in sheep, BMP4 did not influence primordial follicle activation or promote follicle growth (Bertoldo et al., 2014). Different species may account for the differences, but further studies will be needed to explore the truth.

As for BMP7, Lee et al. (2001) showed that rat ovaries treated with BMP7 *in vivo* had decreased numbers of primordial follicles and increased numbers of growth follicles, suggesting that BMP7 may facilitate the transition of follicles from the primordial stage to the pool of growth follicles.

### Anti-mullerian Hormone

The anti-Mullerian hormone (AMH) is a glycoprotein of the TGF-β superfamily and is produced by the granulosa cells of pre-antral and early antral ovarian follicles in several species (Umer et al., 2019). It is generally accepted that AMH inhibits the activation of mammalian primordial follicles (Shahrokhi et al., 2018). An earlier study using *AMH*-knockout mice has already shown that there were more growing follicles and fewer primordial follicles in *AMH*^–/–^ mice compared with the control group (Durlinger et al., 1999). Subsequently, other scientists who cultured mouse ovaries in the absence or presence of AMH noted that cultured ovaries with AMH contained 40% fewer growing follicles compared with control ovaries (Durlinger et al., 2002), and AMH treatment decreased the primordial-to-primary follicle transition (Nilsson et al., 2007). Not just in mice, the role of AMH in the suppression of follicle activation was also verified in bovines (Yang et al., 2017) and humans (Carlsson et al., 2006). Administration of a recombinant human AMH reduced the follicle activation induced by cyclophosphamide in a mouse model, thereby protecting the primordial follicle reserve and improving long-term fertility and reproductive outcomes (Roness et al., 2019).

### Growth Differentiation Factor-9

Growth differentiation factor-9 (GDF-9), a member of the TGF-β superfamily, is secreted by mammalian oocytes (Emori and Sugiura, 2014). Nilsson et al. (2002) reported that GDF-9 promoted the growth of early primary follicles, but not primordial follicles, in cultured rat ovaries. In *GDF-9*-deficient female mice, the primordial and primary one-layer follicles can be formed, but there is a blockage in follicular development beyond the primary one-layer follicle stage, which leads to complete infertility (Dong et al., 1996). These results indicated that GDF-9 mainly activated the growth of primary follicles and might induce the activation of primordial follicles indirectly through the exhaustion of primary follicles. Moreover, oocyte growth was not affected in *GDF-9*-deficient female mice, indicating that GDF-9 functions by activating the granulosa cells while limiting the growth of oocytes in primordial follicles (Dong et al., 1996).

### Basic Fibroblast Growth Factor

Basic fibroblast growth factor (bFGF), also called FGF-2, is mainly situated in the oocytes of early stage rat follicles (Nilsson et al., 2001). In rat ovaries, bFGF was found to decrease the number of primordial follicles and concomitantly increase the amount of developing follicles (Nilsson et al., 2001). Interestingly, the ability of bFGF to induce primordial follicle development needs the presence of KITL (Nilsson and Skinner, 2004). Except for rat ovaries, experiments using the ovaries of goats and humans also suggest that bFGF could activate primordial follicles (Garor et al., 2009; Wang et al., 2014).

### Epidermal Growth Factor

A significant body of work has shown that epidermal growth factor (EGF) is an important regulator of primordial follicle activation in mammals (McNatty et al., 1999; Silva et al., 2004; Adhikari and Liu, 2009; Lu et al., 2015). EGF initiates rat primordial follicle growth by activating proto-oncogene *c-erB2* (Li-Ping et al., 2010) and *c-src* (Du et al., 2012). *c-erB2*, encoding a transmembrane EGF receptor (Coussens et al., 1985; Rajadurai et al., 2018), induces rat primordial follicle activation *via* the downstream PKC and MAPK pathway (Li-Ping et al., 2010). EGF, as the upstream activator of the Src protein, regulates rat primordial follicle growth through the Src→PI3K→PKC→MAPK pathway (Du et al., 2012). But Maruo et al. (1993) demonstrated that the EGF and EGF receptor were observed only in oocytes in the pre-antral follicle stage in humans, but not in primordial follicles. Therefore, more comprehensive studies are needed.

### Leukemia Inhibitory Factor

Leukemia inhibitory factor (LIF) is a pleiotropic cytokine from the interleukin (IL)-6 family regulating various cellular functions (Mathieu et al., 2012). There are several reports on the function of LIF in follicular growth. For example, LIF has been reported to accelerate the primordial-to-primary follicle transition in rat ovaries (Nilsson et al., 2002) and goat ovarian tissues (da Nóbrega et al., 2012) *in vitro*. Yet, Komatsu et al. (2015) discovered that LIF inhibited the growth of mouse primary, secondary, and antral follicles and proved this using the neutralizing antibody *in vitro*. This result is the opposite of previous views and suggests that LIF is likely to coordinate follicular growth in the ovary (Komatsu et al., 2015).

## Conclusion

**FIGURE 2:**
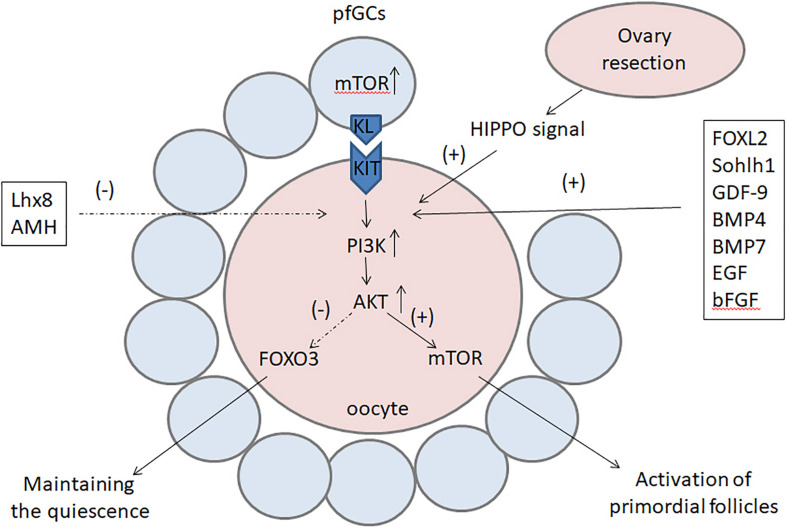
The factors and pathways regulating the activation of primordial follicles. The PI3K–Akt signal is the main transduction pathway in oocytes and plays a key role in the activation of primordial follicles. On the one hand, the PI3K–Akt signal can upregulate mTOR to activate primordial follicles; on the other hand, it can inhibit FOXO3 to locate to the nucleus and prevent it from maintaining the quiescence of primordial follicles. The activation of primordial follicles can also be activated by the mTOR signal in pfGCs and the Hippo signal after ovary resection. Factors such as FOXL2, Sohlh1, NOBOX, GDF-9, BMP4, BMP7, bFGF, and EGF initiate the activation of primordial follicles, whereas Lhx8 and AMH are suppressive factors in this process. *PI3K*, phosphoinositide 3-kinase; *Akt*, serine/threonine protein kinase; *mTOR*, mammalian target of rapamycin; *FOXO3*, forkhead box O3; *FOXL2*, forkhead box L2; *Sohlh1*, spermatogenesis- and oogenesis-specific basic helix-loop-helix-containing protein 1; *NOBOX*, newborn ovary homeobox gene; *GDF-9*, growth differentiation factor-9: BMP, bone morphogenetic protein; *bFGF*, basic fibroblast growth factor; *EGF*, epidermal growth factor; *Lhx8*, LIM homeobox 8; *AMH*, anti-Mullerian hormone.

The activation of primordial follicles is a complex but orchestrated process, which is regulated by multiple factors and pathways (Figure 2). The PI3K–Akt–FOXO3 signaling pathway plays a key role in the maintenance of primordial follicles, while the mTOR signal regulates the activation of primordial follicles. Factors such as FOXL2, Sohlh1, NOBOX, GDF-9, BMP4, BMP7, bFGF, and EGF initiate the activation of primordial follicles, whereas Lhx8 and AMH are suppressive factors in this process. In addition, there have been successes in triggering the activation of primordial follicles *in vitro* to obtain fertilizable oocytes with PI3K stimulators or CDC42 activators in mice.

Although many drugs have not been used in humans due to ethical issues, our growing knowledge of the molecules that control the activation of primordial follicles will provide new but promising methods for obtaining mature eggs, helping women who are unable to have a child because of various ovarian diseases.

## Author Contributions

YC and WY wrote the manuscript and performed all of the necessary literature searches and data compilation. XS and CZ performed the necessary literature searches and data compilation. GS revised the manuscript and gave valuable suggestions. DH designed the review, reviewed it, and approved the submitted manuscript. All the authors contributed to the article and approved the submitted version.

## Conflict of Interest

The authors declare that the research was conducted in the absence of any commercial or financial relationships that could be construed as a potential conflict of interest.
